# Vascular comorbidity is associated with decreased cognitive functioning in inflammatory bowel disease

**DOI:** 10.1038/s41598-023-31160-3

**Published:** 2023-03-15

**Authors:** Ronak Patel, Ruth Ann Marrie, Charles N. Bernstein, James M. Bolton, Lesley A. Graff, James J. Marriott, Chase R. Figley, Jennifer Kornelsen, Erin L. Mazerolle, Md Nasir Uddin, John D. Fisk, Comorbidity and Cognition in Multiple Sclerosis (CCOMS) Study Group, James Bolton, Lesley Graff, Jennifer Kornelsen, Erin Mazerolle, Ronak Patel, Teresa D. Figley, Carl A. Helmick

**Affiliations:** 1 Department of Clinical Health Psychology, Rady Faculty of Health Sciences, Max Rady College of Medicine, University of Manitoba, PZ350-771 Bannatyne Ave., Winnipeg, MB R3E 3N4 Canada; 2 Department of Internal Medicine, Rady Faculty of Health Sciences, Max Rady College of Medicine, University of Manitoba, Winnipeg, MB Canada; 3 Department of Community Health Sciences, Rady Faculty of Health Sciences, Max Rady College of Medicine, University of Manitoba, Winnipeg, MB Canada; 4 Department of Psychiatry, Rady Faculty of Health Sciences, Max Rady College of Medicine, University of Manitoba, Winnipeg, MB Canada; 5 Department of Radiology, Rady Faculty of Health Sciences, Max Rady College of Medicine, University of Manitoba, Winnipeg, MB Canada; 6 Division of Diagnostic Imaging, Winnipeg Health Sciences Centre, Winnipeg, MB Canada; 7 Neuroscience Research Program, Kleysen Institute for Advanced Medicine, Winnipeg Health Sciences Centre, Winnipeg, MB Canada; 8 Department of Psychology, St. Francis Xavier University, Antigonish, NS Canada; 9 Department of Neurology, University of Rochester, Rochester, NY USA; 10 Departments of Psychiatry, Psychology & Neuroscience, and Medicine, Dalhousie University, Halifax, NS Canada; 11 University of Manitoba, Winnipeg, Canada

**Keywords:** Psychology, Gastroenterology

## Abstract

Reports of cognitive impairment in inflammatory bowel disease (IBD) have been mixed. IBD and cardiovascular disease are often co-morbid, yet it remains unknown whether vascular comorbidity confers a risk for decreased cognitive functioning, as observed in other populations. Participants with IBD were recruited from a longitudinal study of immune-mediated disease. Participants were administered a standardized neuropsychological test protocol, evaluating information processing speed, verbal learning and memory, visual learning and memory, and verbal fluency/executive function. Cognitive test scores were standardized using local regression-based norms, adjusting for age, sex, and education. Vascular risk was calculated using a modified Framingham Risk Score (FRS). We tested the association between FRS and cognitive test scores using a quantile regression model, adjusting for IBD type. Of 84 IBD participants, 54 had ulcerative colitis and 30 had Crohn’s disease; mean (SD) age was 53.36 (13.95) years, and a high proportion were females (*n* = 58). As the risk score (FRS) increased, participants demonstrated lower performance in information processing speed (β = − 0.12; 95% CI − 0.24, − 0.006) and verbal learning (β = − 0.14; 95% CI − 0.28, − 0.01) at the 50^th^ percentile. After adjusting for IBD type and disease activity, higher FRS remained associated with lower information processing speed (β = − 0.14; 95% CI − 0.27, − 0.065). Vascular comorbidity is associated with lower cognitive functioning in persons with IBD, particularly in the area of information processing speed. These findings suggest that prevention, identification, and treatment of vascular comorbidity in IBD may play a critical role for improving functional outcomes in IBD.

## Introduction

Inflammatory bowel disease (IBD), including Crohn’s disease (CD) and ulcerative colitis (UC) is a complex chronic immune-mediated disease whereby the gastrointestinal tract becomes inflamed and ulcerated^1^. Canada is among countries with the highest incidence rate of IBD in the world^2^. In 2018, the number of Canadians living with IBD was approximately 270,000 and this number is predicted to rise to 403,000 by 2030^2^. Globally, epidemiological studies have demonstrated a growing prevalence and burden of IBD around the world^3^.

The last decade has seen an increased focus on understanding the role of the gut-brain axis in human disease^4^. This bidirectional communication pathway between the gut and brain involves a complex interplay between neural, immune, and endocrine systems which is thought to have a critical role in the pathophysiology of IBD^5,6^ Stress-inducing conditions are now known to lead to the activation of peripheral and neuronal cell pathways that influence neuroinflammatory mechanisms. Chronic neuroinflammation can lead to neuronal cell death^7^. Increasing attention has been given to how these neuronal changes might affect cognitive functioning in IBD, although data regarding cognitive functioning in persons with IBD remain relatively limited^8^.

Early studies examining the link between IBD and cognition found that persons with IBD exhibited a relative and selective deficit in verbal intellectual functioning (i.e., the ability to reason and solve problems using language) as compared to their own performance-based IQ and to healthy controls^9,10^. However, subsequent studies have not reported this selective deficit in verbal intellectual functioning. Some studies have failed to find any changes in cognitive functioning in persons with IBD^11^, while others have failed to find differences in cognitive functioning between persons with IBD and controls when variables such as concurrent mood disorder and level of education were considered^5^. Several comorbid conditions have been linked to IBD, with a growing number of studies demonstrating that patients with IBD have an increased risk of developing cardiovascular disease^12^ Both conditions reflect chronic inflammatory processes and share certain pathophysiological mechanisms that may influence each other^13–15^. In the general population, cardiovascular disease, including high blood pressure and diabetes, is associated with cognitive decline and is known to affect cognitive functions such as information processing speed (i.e., how quickly information is processed), executive functioning (i.e., higher-order functions such as planning, organization, reasoning, and problem-solving), and learning and memory (i.e., the ability to retain new information)^16^. However, to our knowledge, no study to date has directly examined the effect of comorbid cardiovascular disease and related risk factors on cognitive functioning in persons with IBD.

One measure that simultaneously accounts for multiple cardiovascular conditions and risk factors, while also considering hypertension treatment status and gender, is the Framingham Risk Score (FRS)^17^. Higher FRS scores have been associated with decreased brain volume in population-specific samples such as older adults^18,19^ and more recent findings that reveal lower FRS scores in type 2 diabetes may be associated with better cognitive performance, as compared to those with higher FRS scores^20^. Given the increased co-occurrence of cardiovascular disease in IBD^12^, and inconsistencies in findings of prior studies of cognition in IBD, we aimed to evaluate the association between FRS and cognitive performance in a sample of persons with IBD. We hypothesized that higher FRS in individuals with IBD would be associated with poorer cognitive functioning.

## Methods

### Participants

The source population was participants in a longitudinal study of the effects of psychiatric comorbidity on chronic immune-mediated inflammatory diseases (the ‘IMID’ study) that recruited 247 persons with clinically confirmed IBD^21^. The present study enrolled a subgroup of 84 IBD study participants aged ≥ 18 years, with adequate knowledge of the English language. This subgroup sample reflected a group of participants who were serially approached for participation on the basis of whether they were still participating in the IMID study and had an upcoming annual visit between September 2016 and July 2017, during which funding for the current study was available. Exclusion criteria included comorbid brain tumors or neurodegenerative disorders or contraindications to magnetic resonance imaging, The University of Manitoba Health Research Ethics Board approved the study and all participants provided written informed consent. This research was performed in accordance with relevant guidelines/regulations set out by the University of Manitoba Health Research Ethics Board. As described in detail elsewhere^21^, participants completed validated questionnaires, and underwent standardized clinical assessments conducted by trained personnel. Participants nor the public were involved in the design, conduct, reporting, or dissemination of our research.

### Sociodemographic information and health behaviors

Participants reported sex, date of birth, race and ethnicity (white and non-white), highest level of education attained, annual household income, and marital status. Highest level of education completed was reported as elementary school, junior high school, high school diploma/General Education Diploma (GED), college, technical/trade, university bachelor’s degree, university master’s degree, university doctorate or other. Annual household income was reported as < $15,000, $15,000–29,999, $30,000–49,999, $50,000–100,000, > $100,000 or ‘I do not wish to answer’. Participants who reported ever smoking ≥ 100 cigarettes were classified as smokers^22^. Current smoking status was reported as not at all, some days, or every day. Body mass index (BMI, kg/m^2^) was calculated based on height and weight measured at the study visit.

### Clinical characteristics

We extracted age at symptom onset, age at IBD diagnosis, and type of IBD diagnosis (ulcerative colitis or Crohn’s disease) and current disease-modifying pharmacological agents used (if any) from medical records. Disease-modifying pharmacological agents included thiopurines, methotrexate, TNF-alpha antagonists, ustekinumab, vedoliuzmab, corticosteroids and 5-aminosalicylates (5-ASA). All other medications were recorded by patient interview. We used the Montreal Classification to classify IBD disease course^23^.To assess whether or not there was likely active intestinal inflammation, stool was collected for calprotectin measurement, where a value of ≥ 250 mcg/g of stool was considered active disease^24^.

We used a validated comorbidity questionnaire to capture the number of physical comorbidities that participants had^25^; these comorbid conditions could include chronic lung disease, cancer (breast, colon, lung, skin, and other), migraine, thyroid disease, lupus, osteoarthritis, osteoporosis, fibromyalgia, kidney disease, peptic ulcer, liver disease, and epilepsy. We report physical comorbidities as a count (0, 1, ≥  2).

### Cognition

Our neuropsychological test battery consisted of well-validated measures assessing the major domains of information processing speed (the rate at which information is processed), verbal learning and memory (the ability to learn and retain information that we hear), visual learning and memory (the ability to learn and retain information that we see), and verbal fluency/executive ability (the ability to generate words utilizing different search strategies). We assessed information processing speed using the oral version of the Symbol Digit Modalities Test^26^ (SDMT) verbal learning and memory using the California Verbal Learning Test^27^ (CVLT-II; Trial 1–5 total recall score), visual learning and memory using the Brief Visuospatial Memory Test-Revised^28^ (BVMT-R; summed recall score for all three learning trials), and language and executive abilities using tests of verbal fluency^29^ (letter and animal categories). Neuropsychological tests were administered and scored by research assistants who underwent a comprehensive training protocol led and supervised by a board-certified clinical neuropsychologist (first author R.P.).

### Vascular comorbidity

we focused on hypertension and diabetes because of their associations with disability in other clinical populations and their effects on cognition in the general population^30^. Participants reported these comorbidities using a validated questionnaire^25^. They reported if a physician had diagnosed the comorbidity, and if yes, the year of diagnosis and whether the condition was currently treated.

We augmented the information provided by questionnaire with additional assessments. At the in-person study visit, concurrent with participants’ cognitive assessment, blood pressure was measured once in the seated position using an automatic blood pressure machine. We collected a serum sample to measure hemoglobin A1c (HbA1c). We classified participants as hypertensive^31^ (i.e., any of: self-reported or physician-diagnosed hypertension, use of hypertensive medications, measured BP > 140/90) or not. We classified participants as having diabetes (i.e., any of: self-reported or physician-diagnosed diabetes, use of medications for diabetes, HbA1c > 6.5%^32^) or not. We also classified participants as having hyperlipidemia (self-reported or physician-diagnosed hyperlipidemia or use of lipid-lowering medications) or not. As few participants with any of these conditions reported being untreated, we did not pursue analyses stratifying these conditions by treatment status.

We then used this information to calculate vascular risk for each participant. The Framingham Risk Score (FRS) is a sex-specific weighted index, and incorporates information regarding age, smoking history, hypertension, and diabetes, as well as either lipid status or body mass index^17^. Points are added for factors that increase cardiovascular risk such as a history of smoking and current cardiovascular conditions, while negative points are assigned for factors that are protective^17^. Points for hypertension incorporate measured systolic blood pressure and treatment status (treated or not treated). Because we did not have serum lipid measurements, we used the version of the FRS that relied on body mass index. Age was excluded from the FRS to ensure we did not confound the effect of age on cognition with the effects of vascular risk factors^19^.

### Psychiatric comorbidity

Participants reported symptoms of depression and anxiety using the Hospital Anxiety and Depression.

Scale^33^ (HADS), which is validated for use in IBD populations^34^. The HADS includes 7 items each that assess symptoms of depression (HADS-D) and anxiety (HADS-A) respectively, with total scores ranging from 0 to 21. Participants were classified as to whether they had clinically meaningful symptoms of depression (HADS-D score) or anxiety (HADS-A score). The literature varies regarding the optimal cut-point for the HADS in IBD. Therefore, we employed the more specific cut-point of ≥ 11, which indicates clinically meaningful symptoms of depression and anxiety^35^.

### Statistical analyses

We used descriptive statistics to characterize the study population, including mean (standard deviation), median (interquartile range) and frequency (percent). For the cognitive data, raw test scores were converted to age, sex and education-adjusted z-scores using locally derived regression-based norms^36^. Z-scores of ≤ − 1.5 were classified as impaired. The Wechsler Test of Adult Reading (WTAR), was included to provide an age-, sex-, education-, and ethnicity-adjusted Full-Scale IQ estimate of premorbid intelligence and was used to characterize the sample^37^. We examined Spearman correlations (95% confidence intervals [CI]) between the FRS and cognitive test results. We tested the association between the FRS and cognition using quantile regression. Quantile regression allows the evaluation of a relationship of an independent variable across the full range of a continuous dependent variable rather than its conditional mean and does not require distributional assumptions such as normality or homoscedasticity^38^. In the absence of any a priori information to guide the choice of quantile, we examined the 50th percentile (quantile). The primary outcome (dependent variable) was information processing speed (SDMT), while the independent variable of interest was FRS score (continuous). Secondary analyses were conducted with other cognitive variables of interest (e.g., verbal learning and memory [CVLT], and visual learning and memory [BVMT-R]). We adjusted for IBD type and active disease but did not adjust for psychiatric comorbidity (i.e., HADS-A and HADS-D scores) due to the small number of participants with elevated anxiety/depression scores (see Table 1). To account for the multiple comparisons introduced by using multiple cognitive tests in the regression analysis we applied a Benjamini–Hochberg correction for multiple comparisons for the secondary outcomes, with a false discovery rate of 0.05. Statistical analyses were completed using SAS V9.4 (SAS Institute Inc., Cary, NC).

**Table 1:** Descriptive statistics of the entire IBD sample, ulcerative colitis and Chron's disease (CD) subgroups; Mean (SD).

Characteristic		All (n = 84)	UC (n = 54)	CD (n = 30)		Parametric *P*-value*		Non-Parametric *P*-value**
Age		53.36 (13.95)	52.61 (13.8)	54.71 (14.34)		0.51		0.45
Gender, n (%)	Male	26 (31.0)	15 (27.78)	11 (36.67)		0.40		0.46
	Female	58 (69.0)	39 (72.22)	19 (63.33)				
Race, n (%)	Non-White	9 (10.7)	4 (7.41)	5 (16.67)		0.19		0.27
	White	75 (89.3)	50 (92.59)	25 (83.33)				
Education, n (%)	≤ High School/GED	14 (17.5)	7 (13.73)	7 (24.14)		0.24		0.36
	> High School	66 (82.5)	44 (86.27)	22 (75.86)				
Income, n (%)	< 50,000	17 (20.2)	12 (22.22)	5 (16.67)		0.58		0.66
	> 50,000	62 (73.8)	38 (70.37)	24 (80)				
	Declined to report	5 (6.0)	4 (7.41)	1 (3.33)				
Current Smoker, n (%)	No	73 (86.9)	47 (87.04)	26 (86.67)		0.96		1.00
	Yes	11 (13.1)	7 (12.96)	4 (13.33)				
Other comorbidities, n (%)	0	24 (28.6)	15 (27.78)	9 (30)		0.83		0.84
	1	20 (23.8)	12 (22.22)	8 (26.67)				
	2	40 (47.6)	27 (50)	13 (43.33)				
Current BMI		27.84 (5.92)	26.99 (5.32)	29.39 (6.69)		0.075		0.13
Any disease modifying pharmacotherapy, n (%)	No	25 (29.8)	15 (27.78)	10 (33.33)		0.59		0.63
	Yes	59 (70.2)	39 (72.22)	20 (66.67)				
HADS-D < 11	No	81 (96.40)	53 (98.15)	28 (93.33)		0.26		0.29
HADS-D ≥ 11	Yes	3 (3.60)	1 (1.85)	2 (6.67)				
HADS-A < 11	No	72 (85.70)	48 (88.89)	24 (80.00)		0.26		0.33
HADS-A ≥ 11	Yes	12 (14.3)	6 (11.11)	6 (20.00)				
Active disease^a^, n (%)	No	47 (58.8)	31 (59.6)	16 (57.1)		0.83		
	Yes	33 (41.2)	21 (40.4)	12 (42.9)				
UC Involvement, n (%)	E1: Ulcerative Proctitis		–	3 (10.34)		0.59		0.63
	E2: Left-sided disease		–	14 (48.28)				
	E3: Extensive ulcerative colitis		–	12 (41.38)				
CD Location, n (%)	L1: Terminal Ilium Only		20 (37.74)	–		0.59		0.63
	L2: Colon Only		6 (11.32)	–				
	L3: Small Bowel and Colon		27 (50.94)	–				
CD Behavior, n (%)	B1: Inflammatory		19 (35.19)	–		0.59		0.63
	B2: Structuring		18 (33.33)	–				
	B3: Fistulizing		17 (31.48)	–				
CD: Upper GI involvement, n (%)			–	1.2				
CD Perianal disease, n (%)			–	8.3				

a = 4 missing.

*The parametric *P*-value is calculated by ANOVA for numerical covariates and chi-square test for the categorical covariates.

**The non-parametric *P*-value is calculated by the Kruskal–Wallis test for numerical covariates and Fisher's exact test for categorical variables.

## Results

Out of the 84 participants enrolled, 54 had a diagnosis of UC and 30 had a diagnosis of CD. The sample overall was predominantly white females with the equivalent of a high school education or greater. Over half were currently taking a disease-modifying pharmacological therapy/agent (see Table 1). Generally, participants had relatively intact cognitive functioning with above average overall estimated intellectual functioning (Table 2). Participants generally performed within the average range across all cognitive domains; a similar pattern was observed when scores were separated by IBD subtype (Table 3). Correlational analyses revealed higher vascular comorbidity, as measured by higher FRS, was associated with poorer performance in the areas of information processing speed, verbal learning, visual memory, and verbal fluency (Table 4). In a quantile regression model, higher FRS was associated with lower information processing speed and verbal learning at the 50^th^ percentile (Table 5). After adjustment by IBD subtype and active disease, higher FRS remained associated with lower cognitive functioning for information processing speed (*P*-value = 0.041). The magnitude of the association of higher FRS with lower verbal learning remained similar, with broader confidence intervals observed, but the association was not statistically significant. These results generally remained unchanged when excluding those participants with elevated mood and anxiety symptoms (supplemental table e1).

**Table 2:** Cognitive functioning results (z-scores) for full IBD sample.

FSIQ estimate, mean (SD)	111.02 (6.80)
SDMT, Man mean (SD)	0.23 (1.24)
CVLT verbal learning, mean (SD)	0.57(1.19))
CVLT delayed recall, mean (SD)	0.64 (1.06)
BVMT-R total learning, mean (SD)	− 0.05 (0.88)
BVMT-R delayed recall, mean (SD)	0.37 (1.10)
LNS, mean (SD)	0.01 (1.03)
Verbal fluency, mean (SD)	− 0.11 (1.03)

*FSIQ* Full Scale Intellectual Quotient, *SDMT* Symbol Digits Modality Test, *CVLT* California Verbal Learning Test, 2nd Ed., *BVMT-R* Brief Visualspatial, Test – Revised, *LNS* Letter Number Sequencing Test.

**Table 3:** Cognitive functioning results for UC vs. CD.

	UC	CD
FSIQ estimate, mean (SD)	111.30 (6.34)	110.52 (7.67)
SDMT, Man mean (SD)	0.29 (1.30)	0.12 (1.12)
CVLT verbal learning, mean (SD)	0.70 (1.13)	0.33 (1.28)
CVLT delayed recall, mean (SD)	0.71 (0.99)	0.51 (1.18)
BVMT-R total learning, mean (SD)	0.01 (0.85)	− 0.16 (0.93)
BVMT-R delayed recall, mean (SD)	0.10 (1.09)	− 0.28 (1.09)
LNS, mean (SD)	0.04 (0.95)	0.20 (1.18)
Verbal fluency, mean (SD)	0.20 (0.99)	0.04 (1.12)

*FSIQ* Full Scale Intellectual Quotient, *SDMT* Symbol Digits Modality Test, *CVLT* California Verbal Learning Test, 2nd Ed., *BVMT-R* Brief Visualspatial, Test – Revised, *LNS* Letter Number Sequencing Test.

**Table 4:** Spearman correlation coefficients (95% confidence intervals) between FRS and cognitive functioning z-scores.

	*r-value*	*P*-value
SDMT	− 0.28 (− 0.46, − 0.06)	**0.01**
CVLT-II verbal learning	− 0.25 (− 0.44, − 0.04)	**0.02**
CVLT-II delayed recall	− 0.21 (− 0.40, 0.01)	0.056
BVMT-R total learning	− 0.25 (− 0.44, − 0.04)	**0.02**
BVMT-R delayed recall	− 0.26 (− 0.44, − 0.04)	**0.02**
LNS	− 0.09 (− 0.29, 0.13)	0.42
Verbal fluency	− 0.22 (− 0.41, − 0.01)	**0.04**

Significant values are in bold.

*SDMT* Symbol Digit Modalities Test, *CVLT-II* California Verbal Learning Test-II, *BVMT-R* Brief Visuospatial Memory Test-Revised, *LNS* Letter-Number Sequencing Test.

**Table 5:** Unadjusted and adjusted regression coeffecients (95% confidence intervals) for the association between FRS and cognitive functioning.

Quantile	SDMT	CVLT-II	CVLT-II LD	BMVT-R	BMVT-R DR	LNS	Fluency
*Unadjusted*	*Beta (95%CI)*						
0.5	− **0.12 (**− **0.24,-0.01)**	− **0.14 (**− **0.27, **− **0.01)**	− 0.023 (− 0.13, 0.08)	− 0.091 (− 0.16, − 0.02)	− 0.43 (− 0.11, 0.03)	− 0.026 (− 0.14, 0.09)	− 0.075 (− 0.15,0.001)
	***P* = 0.033**	***P* = 0.035**	*P* = 0.48	*P* = 0.054	*P* = 0.34	*P* = 0.68	*P* = 0.077
*Adjusted for IBD type and disease activity*							
0.5	− **0.14 (**− **0.27, 0.065)**	− 0.11 (− 0.22, − 0.008)	− 0.030 (− 0.15, 0.094)	− 0.073 (− 0.15, 0.003)	− 0.068 (− 0.14, 0.004)	− 0.01 (− 0.14, 0.12)	− 0.083 (− 0.17, 0.004)
	***P* = 0.041**	*P* = 0.072	*P* = 0.63	*P* = 0.055	*P* = 0.059	*P* = 0.87	*P* = 0.058

Significant values are in bold.

Sign using wald test as are the other ones sign using LR test.

*SDMT* Symbol Digit Modalities Test, *CVLT-II* California Verbal Learning Test-II, *LD* Long Delay, *BVMT-R* Brief Visuospatial Memory Test-Revised, *DR* Delayed Recall, *LNS*Letter-Number Sequencing Test.

## Discussion

In this cross-sectional study, we examined the association between vascular risk and cognitive function in a sample of persons with clinically confirmed IBD. Findings of altered cognitive function in persons with IBD have been mixed, and to date no study has directly examined the effect of vascular comorbidity on cognitive function in IBD. However, our findings demonstrate an association between increased vascular risk and decreased cognitive function in IBD. We found that higher vascular comorbidity was correlated with poorer performance in information processing speed, verbal learning, visual memory, and verbal fluency. The main results from our quantile regression analyses revealed that higher vascular comorbidity was predictive of lower information processing speed, and this remained true even after adjusting for IBD type and disease activity.

A growing body of literature suggests IBD is associated with cognitive impairment. In a recent systematic review and meta-analysis^8^, it was shown that persons with IBD, in disease remission, exhibited deficits in overall executive functioning including moderate deficits in working memory, as compared to healthy controls. However, the authors failed to find any differences in learning and recall. Our study reveals that cardiovascular disease may be an important factor mediating the relationship between IBD and cognitive impairment. In non-IBD populations, vascular conditions such as diabetes and hypertension have been associated with poorer cognitive outcomes including an increased risk of developing dementia^39,40^. Meta-analytic findings reveal type 2 diabetes is associated with impairments in motor functioning, executive functioning, processing speed, and verbal and visual memory^41^. Similarly, meta-analytic findings (across 12 studies and 4,076 individuals) have demonstrated significant associations between increasing blood pressure and reductions in episodic memory in older adults who are free of clinical dementia or stroke^39^. In our quantile regression model, we found that increased FRS in IBD is associated with decreased information processing speed, a domain frequently affected by cardiovascular disease. Deficits in cognition such as information processing speed have been associated with increasing levels of disability and reduced functioning in everyday life including decreased occupational functioning^42^.

Several purported pathophysiological mechanisms may underlie decreased cognitive function in IBD and co-occurring cardiovascular disease. Such postulated mechanisms include altered metabolic functioning interfering with neurogenesis in regions such as the hippocampus (a region critical for the facilitation of learning and memory), the expression of pro-inflammatory cytokines leading to neuronal damage, and oxidative stress leading to chronic neuroinflammation and neurodegeneration^13–15^. Pathophysiological changes may also be evident as alterations in brain structure. Indeed, we recently demonstrated that higher vascular comorbidity as indexed by FRS was associated with lower brain volume at baseline, and with greater brain volume loss over time, in persons with multiple sclerosis^19^. Future studies should examine whether vascular comorbidity is associated with similar reductions in brain volume and decreased cognitive function over time in IBD. Systematic investigation of determining effects of vascular morbidity on cognitive functioning has been a critical gap in the IBD literature. Our use of FRS as a summary score of vascular risk reduced the number of comparisons, thereby avoiding the challenges associated with having small numbers of participants affected by a specific comorbidity while also accounting for the frequent co-occurrence of comorbidities associated with increased vascular risk. Nevertheless, there are several limitations to our study. We did not include a non-IBD control group with increased vascular risk, and therefore, we were unable to directly test whether there is an additive or synergistic interaction between IBD and vascular risk on cognitive functioning. Inclusion of a group of individuals with vascular risk factors without IBD would allow us to directly examine to what extent the magnitude of the observed effects differs between persons with and without IBD. Our overall sample size was modest, with more individuals in the UC versus CD group, though after adjusting for IBD type and disease activity, the association between increased FRS and lower information processing speed, remained unchanged. As outlined in Table 5, the confidence intervals associated with each of the regression coefficients generally increased in size, most likely reflecting the smaller sample size of each subgroup. We were unable to examine the effects of psychiatric comorbidity, as mental health concerns were only modestly elevated in this sample, but this allowed us to more readily isolate the effects of vascular morbidity on cognition in IBD. Nevertheless, future studies should aim to recruit larger samples to systematically investigate the influence of these additional comorbid factors on cognition in IBD. Hyperlipidemia was not used to calculate our primary exposure of interest (FRS), but the FRS based on BMI performs similarly. In the current study, blood pressure was measured once in the seated position; however, it is important to consider is that FRS does not solely depend on blood pressure measurement. Studies have shown that ambulatory blood pressure monitoring does not substantially improve risk prediction with the FRS over the average of two measurements^43^.Thus, the impact of misclassification based on a single measurement is likely to be small and biased toward the null. Nevertheless, future studies may benefit from using an average across multiple measurements. We were also unable to evaluate the effects of specific therapies or treatments used to manage vascular risk factors, but the FRS incorporates information regarding the severity of risk factors and treatment status for hypertension. This should be the subject of future work.

Our findings demonstrate that higher vascular risk is associated with lower cognitive function in persons with IBD, specifically with respect to information processing speed and learning and memory. These findings suggest that early prevention, identification, and treatment of vascular conditions in IBD may be essential considerations in the overall clinical management of the disease course as it may lead to improved functional outcomes and an overall increase in quality of life. Future studies should examine the role that treatments specifically targeted at reducing vascular risk have on mitigating cognitive decline in IBD. Future studies of cognition in IBD should also consider the potential roles that vascular risk factors and other comorbid conditions may have on current cognitive functioning as well as changes in cognitive functioning over time.

## Supplementary Information

Supplementary Information.

## Data Availability

The data underlying in this article will be shared on reasonable request to the corresponding author.
