# Supplementary material for: Vascular comorbidity is associated with decreased cognitive functioning in inflammatory bowel disease
Source: Sci Rep. 2023 Mar 15;13:4317. doi: 10.1038/s41598-023-31160-3 (PMC10017678; doi:10.1038/s41598-023-31160-3)
Supplement: Supplementary file 1 — Supplementary Information. [file 41598_2023_31160_MOESM1_ESM.docx]

| ***Supplemental table e1. Analyses excluding individuals with elevated symptoms of depression or anxiety (n = 81)*** | | | | | | | |
| --- | --- | --- | --- | --- | --- | --- | --- |
| Quantile | SDMT | CVLT-II | CVLT-II LD | BMVT-R | BMVT-R DR | LNS | Fluency |
| *Adjusted for IBD type and disease activity* |  |  |  |  |  |  |  |
| 0.5 | -0.16  (-0.26, -0.064) | -0.17  (-0.29, 0.049) | -0.12  (-0.24, -0.005) | -0.081  (-0.16, -0.004) | -0.077  (-0.15, 0.001) | -0.061  (-0.17, 0.047) | -0.089  (-0.17, 0.011) |
|  | **p = 0.002** | **p = 0.006** | **p = 0.041** | **p = 0.040** | p = 0.051 | p = 0.26 | **p = 0.025** |
| Sign using wald test as are the other ones sign using LR test | | | | | | | |
